# Supplementary material for: A Likelihood Approach for Real-Time Calibration of Stochastic Compartmental Epidemic Models
Source: PLoS Comput Biol. 2017 Jan 17;13(1):e1005257. doi: 10.1371/journal.pcbi.1005257 (PMC5240920; doi:10.1371/journal.pcbi.1005257)
Supplement: S1 File — (TAR.GZ) [file pcbi.1005257.s014.tar.gz › HSPH_Online-SI-Revision/MSS10/n10-mild/n10-mild_NEW2_table-4.pdf]

1	newly 3 weeks spec	199.	123.96	{12., 242.}	51.3266	0.526136	newly 3 weeks cumul	583.	393.86	{84., 739.}	46.9777	0.360624	newly 5 weeks spec	174.	84.11	{0., 192.}	60.0402	0.792841	newly 5 weeks cumul	973.	581.1	{84., 1069.}	47.0915	0.396657	newly 54 weeks cumul	2959.	844.03	{84., 1634.}	71.5238	0.725557
2	newly 3 weeks spec	442.	426.76	{235., 617.}	26.629	0.139217	newly 3 weeks cumul	1121.	1145.64	{631., 1649.}	28.9973	0.140435	newly 5 weeks spec	488.	354.88	{242., 463.}	28.2664	0.171779	newly 5 weeks cumul	2106.	1903.89	{1143., 2576.}	22.4525	0.122975	newly 59 weeks cumul	3790.	2917.08	{2367., 3460.}	23.1177	0.126497
3	newly 3 weeks spec	438.	309.92	{81., 613.}	44.3105	0.308077	newly 3 weeks cumul	1008.	820.71	{291., 1478.}	39.9018	0.243961	newly 5 weeks spec	488.	267.87	{44., 469.}	46.2643	0.444986	newly 5 weeks cumul	1997.	1394.68	{436., 2561.}	40.0901	0.274563	newly 62 weeks cumul	4433.	2362.84	{638., 3348.}	46.6988	0.348873
4	newly 3 weeks spec	427.	295.23	{104., 470.}	36.63	0.27527	newly 3 weeks cumul	1020.	798.9	{322., 1248.}	31.9059	0.197038	newly 5 weeks spec	527.	271.71	{57., 436.}	48.4915	0.491108	newly 5 weeks cumul	2089.	1361.88	{497., 2078.}	36.495	0.252875	newly 57 weeks cumul	4089.	2207.27	{599., 3072.}	46.0193	0.342123
5	newly 3 weeks spec	105.	95.55	{56., 132.}	24.3143	0.120662	newly 3 weeks cumul	340.	279.57	{188., 364.}	22.4441	0.117159	newly 5 weeks spec	120.	91.32	{47., 132.}	31.1	0.178805	newly 5 weeks cumul	593.	464.24	{306., 625.}	25.6728	0.138257	newly 37 weeks cumul	2494.	1320.24	{812., 1814.}	47.0634	0.296809
6	newly 3 weeks spec	329.	308.02	{96., 523.}	44.231	0.25103	newly 3 weeks cumul	816.	715.01	{239., 1170.}	39.2782	0.221895	newly 5 weeks spec	353.	365.11	{96., 549.}	37.9972	0.222754	newly 5 weeks cumul	1522.	1430.4	{515., 2274.}	37.8725	0.206991	newly 63 weeks cumul	3219.	3113.59	{1544., 3864.}	17.6673	0.106929
7	newly 3 weeks spec	337.	435.48	{140., 624.}	52.2136	0.25125	newly 3 weeks cumul	914.	1175.88	{394., 1698.}	50.1926	0.222819	newly 5 weeks spec	288.	327.38	{112., 464.}	37.5903	0.240514	newly 5 weeks cumul	1547.	1896.92	{862., 2603.}	42.1422	0.204895	newly 59 weeks cumul	3151.	2710.62	{2059., 3339.}	17.3005	0.138111
8	newly 3 weeks spec	276.	331.94	{134., 490.}	45.3768	0.221624	newly 3 weeks cumul	750.	884.29	{370., 1361.}	43.2973	0.199864	newly 5 weeks spec	242.	307.12	{151., 416.}	43.7603	0.201426	newly 5 weeks cumul	1290.	1523.27	{744., 2208.}	38.7434	0.181872	newly 54 weeks cumul	4074.	2648.83	{1932., 3222.}	34.9821	0.221217
9	newly 3 weeks spec	372.	288.84	{114., 459.}	35.7634	0.24748	newly 3 weeks cumul	848.	714.25	{284., 1116.}	33.2936	0.200194	newly 5 weeks spec	527.	321.94	{114., 472.}	38.9564	0.395502	newly 5 weeks cumul	1906.	1350.77	{591., 2005.}	33.5378	0.238311	newly 58 weeks cumul	4128.	2941.3	{1523., 3785.}	28.7485	0.224652
10	newly 3 weeks spec	221.	80.48	{2., 152.}	64.3077	0.739541	newly 3 weeks cumul	630.	341.14	{67., 668.}	50.981	0.425726	newly 5 weeks spec	246.	41.6	{0., 111.}	83.0894	1.39099	newly 5 weeks cumul	1140.	441.27	{69., 805.}	61.7289	0.567644	newly 55 weeks cumul	2746.	529.88	{69., 979.}	80.7036	0.888889
11	newly 3 weeks spec	268.	618.37	{129., 1257.}	155.847	0.505397	newly 3 weeks cumul	729.	3550.4	{501., 4964.}	402.645	0.723725	newly 5 weeks spec	221.	82.14	{0., 182.}	84.0814	1.31802	newly 5 weeks cumul	1225.	3850.67	{612., 5014.}	232.306	0.585699	newly 60 weeks cumul	2530.	3922.9	{927., 5014.}	80.7099	0.377162
12	newly 3 weeks spec	699.	978.3	{309., 1586.}	68.0687	0.293849	newly 3 weeks cumul	1437.	2221.07	{565., 3978.}	83.6166	0.312908	newly 5 weeks spec	522.	530.04	{186., 1076.}	44.6437	0.237112	newly 5 weeks cumul	2666.	3543.24	{1616., 4862.}	52.0225	0.22438	newly 62 weeks cumul	3704.	4324.02	{3329., 5030.}	23.2937	0.119071
13	newly 3 weeks spec	431.	422.18	{148., 630.}	33.0858	0.19628	newly 3 weeks cumul	1134.	1175.62	{536., 1819.}	35.672	0.191521	newly 5 weeks spec	505.	319.7	{124., 477.}	37.7901	0.267207	newly 5 weeks cumul	2205.	1885.55	{954., 2726.}	25.8934	0.165143	newly 54 weeks cumul	4115.	2741.56	{1978., 3325.}	33.9111	0.202347
14	newly 3 weeks spec	356.	399.56	{296., 522.}	24.3427	0.105175	newly 3 weeks cumul	939.	1039.19	{779., 1295.}	21.8094	0.0961877	newly 5 weeks spec	351.	446.35	{319., 655.}	34.3789	0.140215	newly 5 weeks cumul	1659.	1920.6	{1419., 2554.}	24.8981	0.105846	newly 55 weeks cumul	3922.	4640.41	{3362., 7596.}	28.9671	0.118251
15	newly 3 weeks spec	198.	268.91	{95., 465.}	64.3485	0.268137	newly 3 weeks cumul	512.	676.45	{325., 1131.}	54.4277	0.208274	newly 5 weeks spec	238.	285.58	{72., 453.}	55.7479	0.299868	newly 5 weeks cumul	1009.	1249.49	{558., 2005.}	51.3251	0.216241	newly 57 weeks cumul	4011.	2556.22	{1105., 3443.}	36.2698	0.24792
16	newly 3 weeks spec	383.	403.42	{153., 632.}	39.1906	0.196628	newly 3 weeks cumul	938.	1038.59	{400., 1633.}	40.6322	0.186125	newly 5 weeks spec	435.	349.58	{125., 498.}	27.6506	0.193056	newly 5 weeks cumul	1811.	1783.79	{729., 2609.}	30.7084	0.164664	newly 58 weeks cumul	3950.	2983.54	{1862., 3665.}	24.8192	0.153561
17	newly 3 weeks spec	66.	81.24	{62., 101.}	26.5455	0.0988642	newly 3 weeks cumul	166.	187.87	{151., 221.}	16.8855	0.0666455	newly 5 weeks spec	111.	141.15	{108., 175.}	30.1712	0.110732	newly 5 weeks cumul	390.	436.01	{343., 520.}	16.6282	0.0663895	newly 55 weeks cumul	3319.	7298.71	{7092., 7494.}	119.907	0.341564
18	newly 3 weeks spec	196.	147.63	{36., 299.}	51.2092	0.392652	newly 3 weeks cumul	553.	424.75	{140., 774.}	43.1953	0.282796	newly 5 weeks spec	192.	132.04	{15., 282.}	56.4792	0.557054	newly 5 weeks cumul	970.	696.1	{253., 1362.}	45.8165	0.320789	newly 55 weeks cumul	2464.	1291.86	{290., 2658.}	52.0252	0.449783
19	newly 3 weeks spec	378.	360.63	{145., 542.}	31.7857	0.193821	newly 3 weeks cumul	1189.	1086.48	{491., 1645.}	29.9731	0.166385	newly 5 weeks spec	370.	230.21	{113., 340.}	38.5919	0.273305	newly 5 weeks cumul	1992.	1620.4	{845., 2279.}	25.5733	0.157157	newly 58 weeks cumul	3457.	2188.16	{1490., 2759.}	36.7035	0.220252
20	newly 3 weeks spec	289.	259.83	{81., 496.}	45.6782	0.271914	newly 3 weeks cumul	900.	785.4	{261., 1316.}	37.5289	0.215568	newly 5 weeks spec	356.	197.7	{30., 369.}	48.5337	0.4754	newly 5 weeks cumul	1637.	1218.82	{405., 2151.}	39.3513	0.257504	newly 59 weeks cumul	3969.	1792.56	{408., 2949.}	54.836	0.436684
21	newly 3 weeks spec	420.	476.4	{265., 641.}	31.4	0.12947	newly 3 weeks cumul	1015.	1251.76	{702., 1659.}	35.9567	0.139204	newly 5 weeks spec	456.	415.89	{240., 714.}	31.1557	0.147226	newly 5 weeks cumul	1966.	2127.47	{1313., 2858.}	24.909	0.106158	newly 59 weeks cumul	3646.	3682.63	{2420., 7278.}	34.9937	0.140249
22	newly 3 weeks spec	279.	324.61	{147., 418.}	35.2151	0.171934	newly 3 weeks cumul	788.	860.01	{462., 1139.}	26.5393	0.133192	newly 5 weeks spec	256.	342.77	{117., 459.}	53.7461	0.234501	newly 5 weeks cumul	1343.	1544.95	{820., 1911.}	31.9799	0.153856	newly 55 weeks cumul	3250.	4132.03	{1509., 6306.}	55.5745	0.23023
23	newly 3 weeks spec	399.	747.51	{654., 885.}	88.4787	0.272555	newly 3 weeks cumul	1073.	1672.43	{1435., 1952.}	56.6403	0.192131	newly 5 weeks spec	394.	935.82	{881., 1055.}	138.371	0.3716	newly 5 weeks cumul	1876.	3496.02	{3134., 3986.}	87.2292	0.269997	newly 60 weeks cumul	3098.	7858.46	{6311., 8765.}	153.904	0.395503
24	newly 3 weeks spec	199.	141.94	{25., 274.}	53.3668	0.501329	newly 3 weeks cumul	621.	558.37	{138., 975.}	47.0258	0.323511	newly 5 weeks spec	188.	74.69	{2., 143.}	73.9628	0.891024	newly 5 weeks cumul	1038.	737.47	{175., 1267.}	46.6503	0.377275	newly 56 weeks cumul	2085.	883.47	{177., 1576.}	61.4777	0.568918
25	newly 3 weeks spec	253.	1796.91	{911., 2743.}	612.81	0.842932	newly 3 weeks cumul	550.	3628.2	{1399., 5871.}	562.567	0.781187	newly 5 weeks spec	366.	746.1	{184., 1382.}	127.262	0.375889	newly 5 weeks cumul	1317.	5734.4	{3999., 7784.}	337.408	0.648031	newly 63 weeks cumul	2982.	6310.96	{4761., 8211.}	113.632	0.346507
26	newly 3 weeks spec	272.	113.66	{25., 192.}	62.7721	0.551367	newly 3 weeks cumul	780.	491.42	{138., 850.}	43.7897	0.305986	newly 5 weeks spec	259.	56.36	{0., 113.}	80.	1.31905	newly 5 weeks cumul	1322.	627.99	{145., 1029.}	55.7663	0.434418	newly 54 weeks cumul	2407.	720.96	{145., 1323.}	70.2509	0.64156
27	newly 3 weeks spec	426.	380.09	{107., 645.}	43.5516	0.286622	newly 3 weeks cumul	1154.	1077.63	{389., 1835.}	41.5503	0.228326	newly 5 weeks spec	412.	268.61	{52., 421.}	38.5801	0.37765	newly 5 weeks cumul	2041.	1688.75	{564., 2694.}	36.2288	0.233964	newly 56 weeks cumul	3536.	2266.93	{679., 3261.}	36.0891	0.273408
28	newly 3 weeks spec	120.	77.46	{40., 115.}	37.2333	0.230031	newly 3 weeks cumul	339.	252.02	{157., 339.}	27.8407	0.154458	newly 5 weeks spec	111.	68.44	{28., 112.}	41.4054	0.281608	newly 5 weeks cumul	576.	391.99	{246., 548.}	33.2622	0.194639	newly 34 weeks cumul	2091.	882.08	{446., 1286.}	57.8154	0.407411
29	newly 3 weeks spec	361.	317.56	{135., 485.}	31.7452	0.213498	newly 3 weeks cumul	1007.	905.95	{429., 1336.}	29.8878	0.180231	newly 5 weeks spec	360.	236.4	{49., 402.}	39.1056	0.326696	newly 5 weeks cumul	1758.	1423.89	{639., 2145.}	29.6832	0.197402	newly 57 weeks cumul	3678.	2165.74	{831., 3009.}	41.1164	0.297658
30	newly 3 weeks spec	367.	168.98	{46., 373.}	56.9973	0.554854	newly 3 weeks cumul	970.	594.52	{190., 1078.}	45.6289	0.336801	newly 5 weeks spec	361.	101.09	{3., 237.}	72.4626	0.949105	newly 5 weeks cumul	1701.	830.31	{262., 1585.}	53.769	0.435043	newly 56 weeks cumul	3445.	1119.91	{275., 2201.}	67.4917	0.622284
31	newly 3 weeks spec	288.	575.73	{403., 841.}	103.51	0.304281	newly 3 weeks cumul	670.	1134.78	{822., 1733.}	73.3522	0.232667	newly 5 weeks spec	360.	883.94	{753., 1059.}	149.183	0.406863	newly 5 weeks cumul	1418.	2785.13	{2183., 3620.}	99.9683	0.30575	newly 63 weeks cumul	2824.	7361.3	{5109., 9107.}	160.915	0.401206
32	newly 3 weeks spec	126.	91.17	{59., 126.}	30.0238	0.167958	newly 3 weeks cumul	395.	298.59	{231., 377.}	25.1722	0.133134	newly 5 weeks spec	145.	76.32	{39., 113.}	47.3655	0.312505	newly 5 weeks cumul	687.	459.43	{329., 614.}	33.3261	0.186401	newly 36 weeks cumul	3008.	967.13	{616., 1365.}	67.8481	0.515796
33	newly 3 weeks spec	466.	417.84	{258., 599.}	24.9227	0.149014	newly 3 weeks cumul	1168.	1071.37	{655., 1505.}	25.6892	0.141575	newly 5 weeks spec	533.	378.28	{258., 490.}	29.9137	0.211177	newly 5 weeks cumul	2236.	1862.74	{1220., 2445.}	22.1154	0.135225	newly 57 weeks cumul	4145.	3094.05	{2317., 3596.}	26.7264	0.147915
34	newly 3 weeks spec	301.	154.75	{33., 288.}	55.5249	0.43006	newly 3 weeks cumul	838.	513.77	{143., 897.}	45.0871	0.321125	newly 5 weeks spec	335.	106.75	{9., 245.}	71.4478	0.781962	newly 5 weeks cumul	1525.	753.13	{216., 1294.}	53.7856	0.409596	newly 57 weeks cumul	3835.	1083.54	{320., 2129.}	71.746	0.655144
35	newly 3 weeks spec	430.	383.16	{177., 535.}	24.9116	0.13995	newly 3 weeks cumul	1171.	1182.04	{740., 1644.}	26.1383	0.125297	newly 5 weeks spec	358.	250.32	{115., 344.}	30.838	0.217165	newly 5 weeks cumul	1960.	1751.52	{1050., 2333.}	21.7622	0.118613	newly 57 weeks cumul	3634.	2357.46	{1714., 2922.}	35.1277	0.205266
36	newly 3 weeks spec	452.	325.15	{65., 573.}	40.4624	0.308682	newly 3 weeks cumul	1197.	933.48	{253., 1551.}	38.0602	0.250009	newly 5 weeks spec	398.	238.03	{38., 408.}	43.6558	0.452046	newly 5 weeks cumul	2080.	1456.79	{412., 2376.}	37.4736	0.274009	newly 59 weeks cumul	3700.	2090.18	{589., 3063.}	43.5086	0.332796
37	newly 3 weeks spec	333.	501.31	{196., 795.}	70.3393	0.269421	newly 3 weeks cumul	782.	1238.02	{375., 2022.}	76.9923	0.269629	newly 5 weeks spec	357.	407.91	{196., 553.}	34.7535	0.186868	newly 5 weeks cumul	1618.	2116.75	{858., 2934.}	48.9981	0.2114	newly 62 weeks cumul	2716.	3189.43	{2780., 3656.}	22.7861	0.109223
38	newly 3 weeks spec	369.	588.12	{544., 630.}	59.3821	0.201785	newly 3 weeks cumul	949.	1414.76	{1341., 1495.}	49.079	0.173011	newly 5 weeks spec	348.	731.86	{686., 774.}	110.305	0.322311	newly 5 weeks cumul	1684.	2830.36	{2698., 2947.}	68.0736	0.225227	newly 58 weeks cumul	4183.	7555.74	{7467., 7645.}	80.6297	0.256767
39	newly 3 weeks spec	526.	365.03	{97., 610.}	39.135	0.352599	newly 3 weeks cumul	1168.	958.7	{239., 1596.}	38.7757	0.262605	newly 5 weeks spec	460.	285.04	{91., 428.}	39.5087	0.343653	newly 5 weeks cumul	2156.	1583.57	{461., 2504.}	34.2546	0.259026	newly 59 weeks cumul	3091.	2540.49	{1280., 3178.}	19.4581	0.162418
40	newly 3 weeks spec	95.	89.58	{58., 121.}	21.3263	0.100797	newly 3 weeks cumul	322.	281.18	{200., 367.}	19.6149	0.0980118	newly 5 weeks spec	79.	76.65	{39., 115.}	29.4051	0.138334	newly 5 weeks cumul	527.	443.06	{314., 571.}	22.2125	0.113688	newly 32 weeks cumul	1882.	1038.14	{517., 1473.}	44.8385	0.289741
41	newly 3 weeks spec	565.	310.99	{89., 571.}	49.1381	0.397758	newly 3 weeks cumul	1036.	717.94	{237., 1267.}	42.6371	0.291266	newly 5 weeks spec	573.	358.26	{78., 583.}	39.5323	0.366389	newly 5 weeks cumul	2224.	1432.85	{507., 2411.}	40.1407	0.298703	newly 63 weeks cumul	3132.	2883.68	{1218., 3573.}	19.2829	0.149317
42	newly 3 weeks spec	232.	241.23	{66., 442.}	48.1078	0.321314	newly 3 weeks cumul	680.	705.63	{320., 1156.}	38.8985	0.210553	newly 5 weeks spec	278.	198.36	{17., 390.}	46.4604	0.460678	newly 5 weeks cumul	1255.	1127.51	{379., 1926.}	36.5315	0.234631	newly 55 weeks cumul	2911.	1896.74	{423., 2979.}	37.1178	0.305641
43	newly 3 weeks spec	534.	507.21	{185., 776.}	39.3052	0.256175	newly 3 weeks cumul	1357.	1179.6	{390., 1868.}	39.6094	0.254019	newly 5 weeks spec	396.	468.85	{258., 642.}	35.6692	0.190862	newly 5 weeks cumul	2311.	2172.47	{1051., 3000.}	29.7503	0.187012	newly 61 weeks cumul	3391.	3394.9	{2952., 3736.}	9.87201	0.0641404
44	newly 3 weeks spec	300.	361.33	{163., 506.}	40.01	0.1615	newly 3 weeks cumul	794.	927.78	{501., 1327.}	35.2443	0.14304	newly 5 weeks spec	309.	365.97	{177., 530.}	41.466	0.183184	newly 5 weeks cumul	1422.	1667.46	{875., 2190.}	35.7792	0.147478	newly 57 weeks cumul	3738.	3711.26	{1980., 6595.}	42.8165	0.196074
45	newly 3 weeks spec	534.	445.06	{41., 797.}	50.7753	0.423117	newly 3 weeks cumul	994.	886.23	{146., 1646.}	49.6288	0.338423	newly 5 weeks spec	717.	616.64	{67., 1168.}	48.0948	0.399428	newly 5 weeks cumul	2450.	2079.15	{307., 3717.}	46.2984	0.349863	newly 64 weeks cumul	4278.	4530.85	{3045., 6920.}	32.8132	0.183964
46	newly 3 weeks spec	331.	452.17	{165., 708.}	56.3716	0.272043	newly 3 weeks cumul	825.	1139.31	{447., 1764.}	56.8739	0.242296	newly 5 weeks spec	370.	388.7	{193., 542.}	28.8	0.200129	newly 5 weeks cumul	1578.	1979.79	{816., 2898.}	44.2972	0.218159	newly 61 weeks cumul	3207.	3009.02	{2226., 3652.}	14.2027	0.120211
47	newly 3 weeks spec	416.	346.92	{251., 442.}	21.3462	0.119267	newly 3 weeks cumul	828.	754.51	{539., 964.}	19.0592	0.0988906	newly 5 weeks spec	448.	566.44	{391., 719.}	33.6429	0.130779	newly 5 weeks cumul	1770.	1780.72	{1360., 2153.}	15.8079	0.0744135	newly 61 weeks cumul	4274.	6737.79	{3726., 7854.}	63.8917	0.211811
48	newly 3 weeks spec	360.	870.27	{291., 1337.}	153.758	0.443459	newly 3 weeks cumul	945.	2338.55	{699., 4023.}	162.251	0.408277	newly 5 weeks spec	310.	376.34	{131., 598.}	56.5677	0.279278	newly 5 weeks cumul	1682.	3315.94	{1433., 4646.}	108.105	0.335799	newly 58 weeks cumul	3197.	3830.05	{2733., 4728.}	31.0913	0.153739
49	newly 3 weeks spec	197.	86.1	{8., 194.}	59.4518	0.665528	newly 3 weeks cumul	473.	307.09	{72., 610.}	49.1734	0.362906	newly 5 weeks spec	183.	56.69	{0., 130.}	70.9891	1.13959	newly 5 weeks cumul	887.	435.37	{86., 846.}	54.3732	0.465476	newly 54 weeks cumul	3344.	558.69	{86., 1084.}	83.2928	0.940496
50	newly 3 weeks spec	233.	164.62	{35., 315.}	46.9099	0.386957	newly 3 weeks cumul	705.	579.16	{204., 964.}	36.1702	0.230028	newly 5 weeks spec	204.	100.71	{10., 222.}	57.3186	0.641958	newly 5 weeks cumul	1157.	811.61	{292., 1384.}	41.3838	0.289901	newly 54 weeks cumul	2093.	1152.44	{352., 1960.}	46.8686	0.375602
